# Social lives of bacteria as revealed through CLASI-FISH

**DOI:** 10.1042/EBC20250042

**Published:** 2026-09-02

**Authors:** Jessica L. Mark Welch

**Affiliations:** ADA Forsyth Institute, 100 Chestnut Street, Somerville, MA 02143, U.S.A.

**Keywords:** Biofilm, biogeography, confocal microscopy, Gut microbiome, Marine microbiome, Oral microbiome

## Abstract

The spatial organization of microbiomes illuminates their structure, function, and relationship to their host. Using fluorescence spectral imaging to discriminate up to 16 fluorophores and using combinations of probes to generate unique spectral signatures, combinatorial labeling and spectral imaging-fluorescence *in situ* hybridization (CLASI-FISH) has been deployed to analyze spatial organization in oral, gut, and marine microbiomes. In dental plaque, imaging revealed the structural role of *Corynebacterium matruchotii* in organizing the plaque biofilm and revealed previously unrecognized complexity in dental plaque corncob structures. Tongue dorsum biofilms showed a patchy organization around a core of host epithelial cells projecting from the tongue surface, with anaerobes located near the core and oxygen-tolerant taxa near the surface. Taxa that are prominent in these tongue dorsum communities can reduce nitrate to nitrite and thus may play an important role in human nitrate metabolism. In contrast with the highly structured organization of oral biofilms, analysis of the gut microbiome by CLASI-FISH showed a mixed community, indicating that the rate of mixing in the gut is high enough to overcome the tendency of bacterial replication to generate single-taxon patches. Application of CLASI-FISH to blades of kelp showed a dense biofilm with clusters of cocci near the kelp surface, bacteria invading the kelp tissue, and rod-shaped and filamentous bacteria extending into the water column. Collectively, visualizing the spatial organization of host-associated microbiomes reveals spatial relationships among taxa and between microbes and host and serves to generate predictions about the dynamics of the host–microbiome interaction.

## Introduction

One of the great discoveries of recent decades is the astonishing richness and complexity of the microbial world. Habitats such as the ocean, the soil, and the bodies of animals and plants are colonized by many hundreds of species of microbes. Microbes are the foundation of food webs and nutrient cycling. Host-associated microbial communities, or microbiomes, carry out essential physiological functions for their hosts. Yet we are only beginning to understand how these complex microbial communities work. What are the roles of the different members of the community, how do the members interact, and how do the identities and characteristics of individual members affect the function of the community as a whole?

The spatial structure of a microbial community provides important information about the interactions and functional relationships of its members. Bacteria interact with one another both via direct contact and by exchange of nutrients and other molecules by diffusion. Adjacency relationships—who is next to whom—therefore provide information about functional relationships—who is likely to be interacting with whom. The technique of *in situ* hybridization was developed to identify nucleic acids and the bacteria that contain them in their natural context, ‘*in situ’* in their original site, thereby preserving this valuable spatial and structural information.

The present article describes the development of a technique called CLASI-FISH, combinatorial labeling and spectral imaging-fluorescence *in situ* hybridization, and its application to discover the organization of microbes in samples as diverse as human dental plaque, bacteria on the human tongue, the microbiota of the mouse colon, and the surface of marine algae. In each of these environments, CLASI-FISH shows whether the bacteria are mixed or spatially structured, identifies which subsets of taxa are found together, and reveals structures and relationships that lead to hypotheses about the roles and interactions of taxa with their host and with one another in these diverse microbial communities.

## Foundations of rRNA-targeted FISH and the development of CLASI-FISH for identifying bacteria in context

A barrier for understanding spatial organization in bacterial communities is that many kinds of bacteria are morphologically similar to one another; there may be hundreds of kinds of bacteria in a sample, but only a few basic shapes: cocci (spheres), rods, and spirals. Many bacteria are pleomorphic, adopting different shapes depending on the growth conditions. To overcome this barrier, fluorescence *in situ* hybridization (FISH) targeting ribosomal RNA (rRNA) was developed to differentiate types of bacteria from one another.

*In situ* hybridization, a strategy for detecting nucleic acid inside a cell using a labeled, complementary nucleic acid as a probe, was developed in the late 1960s independently by three teams of scientists at Yale University, the University of Edinburgh, and the University of Rome [1–4]. The foundations for identifying bacteria with FISH were laid in the 1970s with the realization that the 16S rRNA gene can be used to identify bacteria and determine their relationships [5]. The use of fluorescently tagged probes targeting rRNA to visualize and identify bacteria in the environment and in human and animal microbiomes began in the 1980s. An advantage of targeting rRNA is that there are hundreds and even thousands of ribosomes per cell. Fluorescent probes can bind to each of them, providing a natural amplification of the fluorescent signal. Using this principle, the first FISH targeting bacterial rRNA was published in 1989 [6]. Over the next decades, protocols were developed and probes designed to target large groups of bacteria as well as individual bacterial species of interest [7–15]. These pioneering works used FISH to investigate the localization of one or two taxa at a time within complex communities or tissues.

The goal of CLASI-FISH was to identify not just one or a few types of bacteria, but all the bacteria in the sample simultaneously, by labeling each taxon with a combination of fluorophores to create large numbers of spectral signatures. In standard FISH, fluorescence signals are generally distinguished using a microscope with band-pass filters. These filters are designed to transmit light only in a certain region (or band) of the spectrum, typically allowing the user to differentiate fluorescent signals in the blue, green, red, and far-red parts of the spectrum.

The technical innovation of CLASI-FISH was to deploy fluorescence spectral imaging to differentiate as many as 16 fluorophores [16,17]. Many fluorophores with different characteristic excitation and emission spectra are commercially available and can be used to label short DNA probes for FISH. Therefore, limits on the number of different probes used in an experiment are generally set not by the available fluorophores but by the imaging equipment. Microscopes equipped for spectral imaging eliminate bandpass filters from the light path and instead use a prism, a diffraction grating, or other technology to separate the emitted light by wavelength and record the spectrum of light making up each pixel in the image [18]. The spectrum from each pixel can then be decoded, and the bacterium identified, by expressing the measured spectrum as a weighted sum of each of the fluorophores using a matrix algebra operation known as linear unmixing.

In the first demonstration of spectral imaging applied to bacterial FISH [16], Gary Borisy, Alex Valm, and their colleagues labeled different tubes of the bacterium *Escherichia coli* with 8 fluorophores in all possible binary combinations, resulting in 28 distinct labels that they were able to discriminate using spectral imaging and linear unmixing. They named this technique CLASI-FISH for the combinatorial labeling (because the spectral signature of each bacterial type is made up of combinations of fluorophores) and spectral imaging, which is used to visualize the signal. They then deployed the technique to differentiate 15 taxa in dispersed dental plaque using binary combinations of 6 fluorophores. In a later proof of principle, they deployed 16 fluorophores to label tubes of *E. coli* with 120 different binary combinations of probes and were able to discriminate all 120 types [17]. Distinguishing all 16 fluorophores, in practice, depended on capturing information from both the excitation and the emission spectra of the fluorophores and was helped by using a simple experimental system, many differently labeled cells from a pure culture of the same bacterium.

In principle, more labels could be created by using ternary and other higher-level combinations of probes. In practice, real-world experiments with samples of bacteria from the environment are more complex because of multiple factors. These factors include autofluorescence from the sample; differing intensity of signal from different taxa or different cells of the same taxon due to differences in target accessibility, ribosome content, and probe hybridization efficiency; and the fact that a single pixel in the image can contain signal from two or more adjacent bacterial cells, rendering combinatorial signals difficult to interpret. As more fluorophores are included in the experiment, spectral overlap becomes greater and linear unmixing correspondingly more difficult. Therefore, the most robust results were obtained using fewer fluorophores and simpler combinations. For this reason, most CLASI-FISH experiments to date have been done with simpler combinations of probes.

The present essay describes how CLASI-FISH has been used to examine the spatial organization of bacteria living in the human mouth, in the mouse colon, and on the surface of marine organisms such as kelp. These diverse samples are good targets for CLASI-FISH because they are colonized by multi-species communities of bacteria growing to high density, up to 10^11^ bacteria per milliliter, such that many bacteria can be visualized in the same microscopic field of view. References in the following sections to bacterial agglomerations in the mouth and on kelp as ‘biofilms’ are meant not in the technical sense that their adhesive extracellular matrix has been identified and analyzed, but purely in the sense that they are a dense aggregate of microbes that must adhere to the substrate or one another to avoid being swept away by the flow of saliva or seawater. The goal of the present essay is to show the value of CLASI-FISH and similar techniques in illuminating the biology of these fascinating communities.

## Social life in dental plaque in health

**Figure 1:**
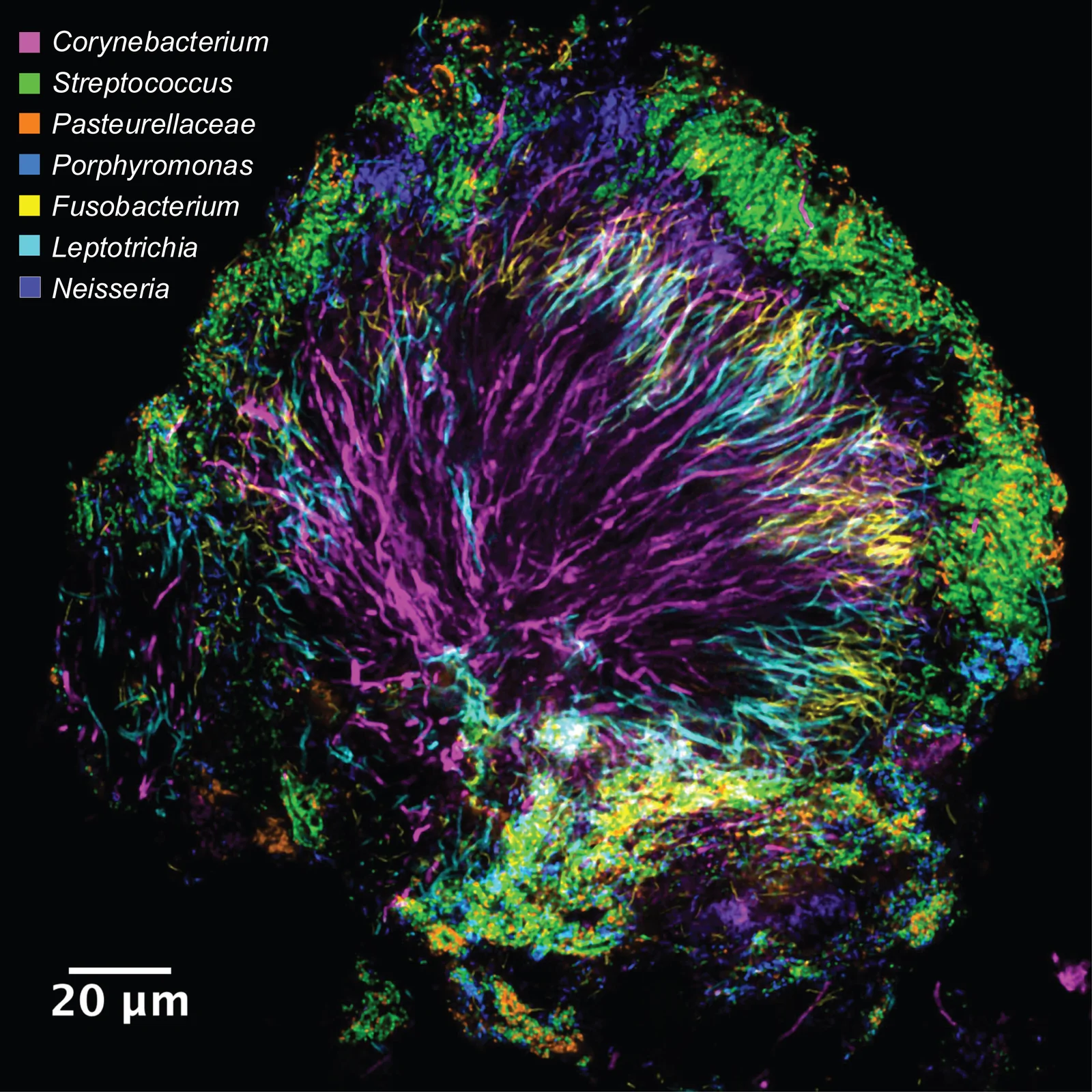
Spatial organization of dental plaque provides clues to taxon-taxon interactions In this ‘hedgehog’ structure from dental plaque, filaments of *Corynebacterium* sp. radiate outward from the center, forming the scaffold of the structure. *Streptococcus*-rich corncobs form an outer shell, and anaerobes inhabit the interior. Modified from [19].

CLASI-FISH images of dental plaque demonstrate why imaging the spatial structure of a microbial community provides clues to how the bacteria interact. Samples of dental plaque from healthy volunteers, scraped from the teeth using toothpicks and imaged with sets of up to 10 probes targeting different genera and families of oral bacteria, showed complex and highly structured communities called ‘hedgehogs’ for their many radially oriented spikes [19]. One such structure is shown in Figure 1. At the center of this structure is a clump of filaments of the taxon *Corynebacterium matruchotii*, shown in magenta. This taxon is one of a few dozen bacterial species that are found in dental plaque with a prevalence of near 100% and an average abundance of 1% or more, but as this taxon was not a pathogen, it had previously attracted relatively little scientific attention. However, its position in the biofilm, as revealed by CLASI-FISH, indicated that it occupied an important role as the foundational member of the structure: the taxon that creates a spatial structure that then generates habitat for other organisms. Around the periphery of the hedgehog, occupying the outermost 20 to 30 μm in the image in Figure 1, were ‘corncob’ structures, consisting largely of streptococci (shown in green) bound to the tips of the *C. matruchotii* filaments. Inside this outer ring of corncobs was an inner zone occupied by cells of the genera *Fusobacterium* and *Leptotrichia*, whose members are generally anaerobes or prefer low-oxygen environments. Looking at the structure, therefore, one could hypothesize that the streptococci at the surface consumed most of the available oxygen, generating a lower-oxygen zone toward the interior of the plaque fragment, thereby creating a suitable habitat for cells of *Fusobacterium* and *Leptotrichia*. Thus, the spatial organization of the biofilm provided clues as to the ways in which its participants interact and influence one another.

**Figure 2:**
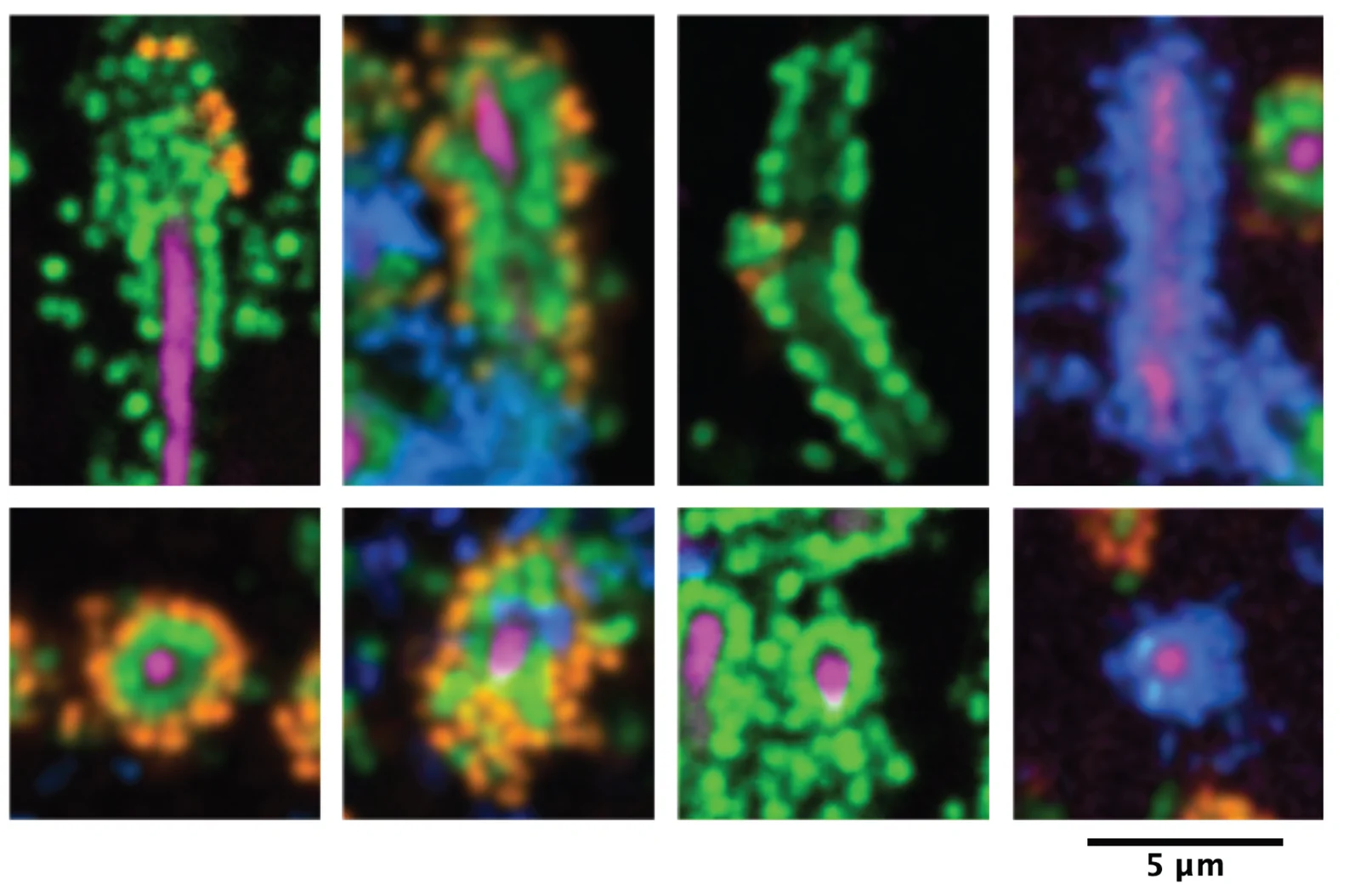
CLASI-FISH shows that dental plaque 'corncob' structures are more complex than previously realized A closer view of corncobs showing streptococci (green) and *Porphyromonas* sp. (blue) bound to the central *Corynebacterium* filament (magenta). An outer layer of Pasteurellaceae (orange) was sometimes present. Each panel shows a different corncob; images in the top row appeared in longitudinal section, and those in the bottom row are in cross section. Modified from [19].

Imaging dental plaque corncob structures with probes targeting different families, genera, and species of bacteria showed that corncobs were more complex than had previously been understood, but they nonetheless contained only a limited subset of the species in dental plaque. The layer of cells directly adjacent to the *Corynebacterium* filament contained not only streptococci but also cells from the genus *Porphyromonas* (Figure 2 and [19]). This finding was unexpected, as the most well-studied organism from the genus *Porphyromonas*, *Porphyromonas gingivalis*, is a strict anaerobe and unlikely to live in the surface, oxygenated layer of plaque where corncobs are found. While *P. gingivalis* is rare in healthy supragingival plaque, two other, little-studied, *Porphyromonas* species (*Porphyromonas pasteri* and *Porphyromonas catoniae*) are common, and this localization suggests that at least one of them is oxygen-tolerant. An outer layer of cells in the corncob, bound to the cells of the inner layer, was composed of bacteria from the family Pasteurellaceae. Using additional probes for more detailed identification of the streptococci revealed that not only *Streptococcus cristatus*, but also cells of *Streptococcus mitis* or *Streptococcus oralis* (difficult to distinguish from one another by FISH), were frequently found in corncobs [20]. However, other abundant plaque streptococci were not observed in corncobs. Thus, the corncob structure appeared to be composed of at least five taxa: *C. matruchotii*, *S. cristatus*, *S. mitis* or *S. oralis*, a species of *Porphyromonas*, and a species of Pasteurellaceae; but other streptococci and other porphyromonads that live in dental plaque were not found in the corncob but were found elsewhere in plaque. Thus, imaging of corncobs showed that dental plaque bacteria are neither arbitrarily distributed nor interchangeable; different species even within the same genus occupy distinct microhabitats within dental plaque.

Outside of the hedgehog structure, other taxon–taxon interactions suggested that both positive interactions (such as nutrients) and negative ones (such as toxins) influence the likelihood that two taxa will be found close to one another. An investigation focused on the interaction between two bacteria abundant in dental plaque, *Haemophilus parainfluenzae* and bacteria of the *S. mitis*/*oralis* group, showed that most *H. parainfluenzae* cells were located within a few micrometers of an *S. mitis*/*oralis* cell, but that density of *H. parainfluenzae* decreased in regions of the biofilm that had high numbers of streptococci. Both observations can be explained by taxon–taxon interaction, as streptococci produce hydrogen peroxide, which can inhibit the growth of *H. parainfluenzae*, but streptococci also produce nicotinamide adenine dinucleotide, an essential metabolite that *H. parainfluenzae* cannot produce and must obtain from its environment [21]. Thus, the observed positioning of *H. parainfluenzae* in the plaque biofilm, close to scattered cells but not large clusters of *S. mitis*/*oralis*, reflects the molecular interactions between them.

## Social life in dental plaque gone bad: cross-kingdom interactions

When two different microbes get together, the combination can have emergent properties that neither organism had by itself. A striking example of this is the combination of streptococci with *Candida albicans*. The fungus *C. albicans* is commonly found in the mouth, usually in low numbers, and it can grow in a yeast form, as round cells, or as long, thin hyphae. In the healthy mouth it is more likely to be growing as a yeast. But when combined with *S. gordonii*, *C. albicans* forms more hyphae, generating a more robust biofilm, and generates a sticky extracellular polymeric matrix that protects the streptococci from antibiotics [22,23].

When *C. albicans* grows together with *S. mutans*, an acid-producing bacterium associated with dental caries (the disease that causes tooth cavities), emergent properties of the combination are more severe. These assemblages attach more strongly to teeth than assemblages composed of *C. albicans* and *S. gordonii* and are more resistant to the antiseptic chlorhexidine [24]. But fluorescence imaging showed a more startling emergent property: as *C. albicans* and *S. mutans* grew, the elongating hyphae of the *Candida* lifted the edge of the assemblage off the surface and then touched down some distance away. The assemblage essentially walked along the surface, spreading much faster and causing greater damage to the teeth than either organism could do alone [24].

The effect of taxon–taxon interactions on the biology of individual taxa and the biofilm as a whole suggests that one promising strategy for preserving or restoring health in the oral microbiota could be to target the interactions rather than the taxa themselves. The growth of beneficial taxa could potentially be encouraged by supplying growth-promoting factors or nutrients normally secreted by neighboring bacteria. Harmful taxa could potentially be neutralized by disrupting their synergistic interactions with neighbors, such as the *C. albicans*–*S. mutans* interaction, perhaps by identifying and blocking the molecules responsible for adhesion between the taxa. Such possibilities represent a rich avenue for future research in oral microbiology.

## Social life on the tongue

**Figure 3:**
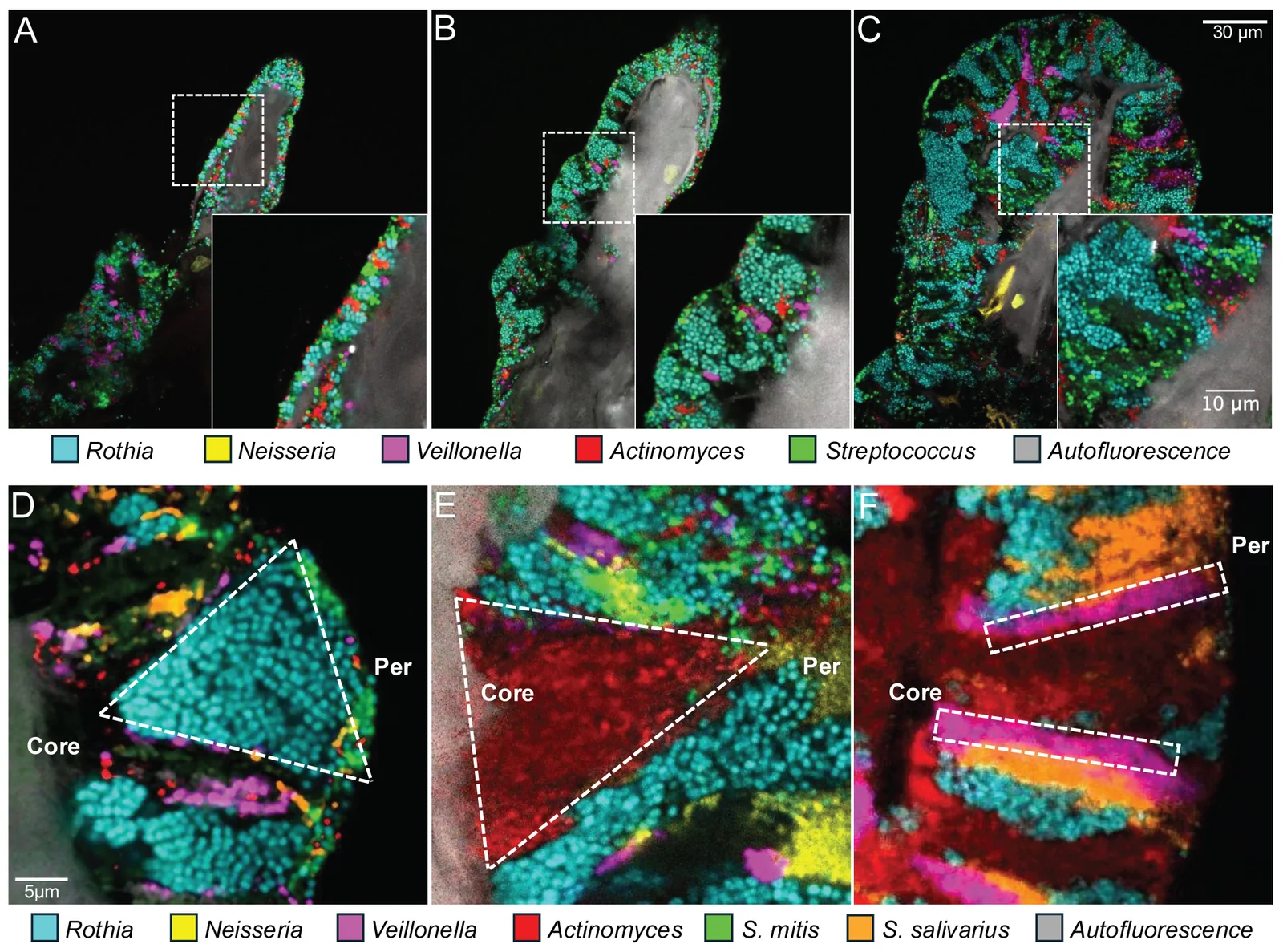
CLASI-FISH imaging shows that cells scraped from the tongue are coated with biofilms of varying thickness Bacterial biofilms scraped from the surface of the human tongue. A central core of epithelial cells (gray) can be coated by a thin biofilm (**A**) or a thicker one (**B,C**). Bacteria form patches that can become wider toward the perimeter (**D**), wider toward the core (**E**), or grow in thin stripes (**F**). Scale bars in panel (C) and panel (C) inset apply to panels (A–C) and their insets; the scale bar in panel (D) applies to panels (D–F). Modified from [26].

DNA sequencing studies show that the bacteria that live abundantly on the tongue are different from those in dental plaque [25], so perhaps it is not a surprise that imaging revealed a distinctly different structure of bacterial communities on the tongue compared with the teeth. Material scraped from the dorsal (top) surface of the tongue and imaged with CLASI-FISH revealed complex consortia organized around a central core of human epithelial cells (Figure 3 and [26,27]). The dorsal surface of the human tongue is populated by numerous filiform papillae, and electron micrographs show that these filiform papillae have projecting hair-like structures that become heavily colonized by bacteria [28]. Images of tongue consortia visualized by CLASI-FISH likely represented a detailed view of these bacteria-coated structures.

Images showed that the central core of epithelial cells was covered by a layer of bacteria that was thin in some examples and thick in others. Arranging these static images in a series suggested a progression from a thinly colonized surface to a gradually thickening biofilm (Figure 3). Some bacteria formed patches that became wider toward the perimeter of the consortium, indicating that these bacteria outcompeted their neighbors for space as the consortium grew or that they thrived in the high-oxygen environment near the surface of the consortium. Other patches, such as the facultatively anaerobic *Actinomyces* spp., became wider toward the epithelial-cell core of the consortium, implying that these bacteria were outcompeted or that they thrived and replicated in the deeper layers of the consortium once the environment became sufficiently anoxic.

Other bacteria were seen forming stripes adjacent to one another, suggesting dependence of the taxa on one another. Waste products for one taxon can be food for another taxon, and organisms benefit from being directly adjacent to their food source. Streptococci take up sugars and convert them into lactic acid, which to the streptococci is a waste product. To surrounding bacteria such as *Veillonella* spp., however, it is a growth-promoting nutrient [29]. The stripes of *Veillonella* sp. adjacent to streptococci in the CLASI-FISH image shown in Figure 3 may be the result of these two taxa thriving when they are adjacent to one another, a spatial relationship that suggests a close metabolic interaction.

The spatial organization of the bacterial biofilm on the tongue relative to the host likewise suggests functional interaction, in this case between host and microbiota. Frequent exfoliation of the epithelial cells at the core of the biofilm is suggested by the fact that tongue microbes are shed into saliva and that entire clusters, including the epithelial cell core, are readily dislodged by gentle scraping. The rate of exfoliation, the rate at which hairs of the papillae are shed and new hairs become available for colonization, would set a limit on the thickness of the bacterial biofilm and therefore the balance between oxygen-tolerant and anaerobic taxa. Thus, we hypothesize that the rate of exfoliation is a mechanism by which the overall structure and composition of the tongue biofilm can be influenced by the physiology of the human host. Although it is not yet possible to image the dynamics of the growth and shedding of these consortia, static imaging nonetheless serves as a hypothesis-generating tool, making predictions about growth dynamics and the impact of host physiology on microbiome structure.

Diet and oral hygiene practices of the human host are also likely to have strong effects on the structure and organization of the tongue biofilm. Nitrate is a nutrient that has attracted special attention as a modulator of both microbial community structure and host physiology. Consuming a meal rich in vegetable nitrate results in a drop in blood pressure, a beneficial effect that depends on the oral microbiota and can be interrupted by frequent use of antiseptic mouthwash [30,31]. Cleaning one’s tongue appears to shift the composition of the oral microbiota toward a community that processes nitrate more effectively [30]. These intriguing interactions indicate that both diet and oral hygiene can have important effects on microbial and host physiology. Thus, bacterial social life on the tongue is shaped by the environment—including the behavioral environment—that the host presents for those bacteria. In future, CLASI-FISH could be used to show how the spatial relationships of tongue bacteria shift when the host cleans the tongue or consumes different amounts of nitrate, information that could be used to develop probiotics or recommendations for diet or hygiene to support this beneficial microbial function.

## Social life in the gut

**Figure 4:**
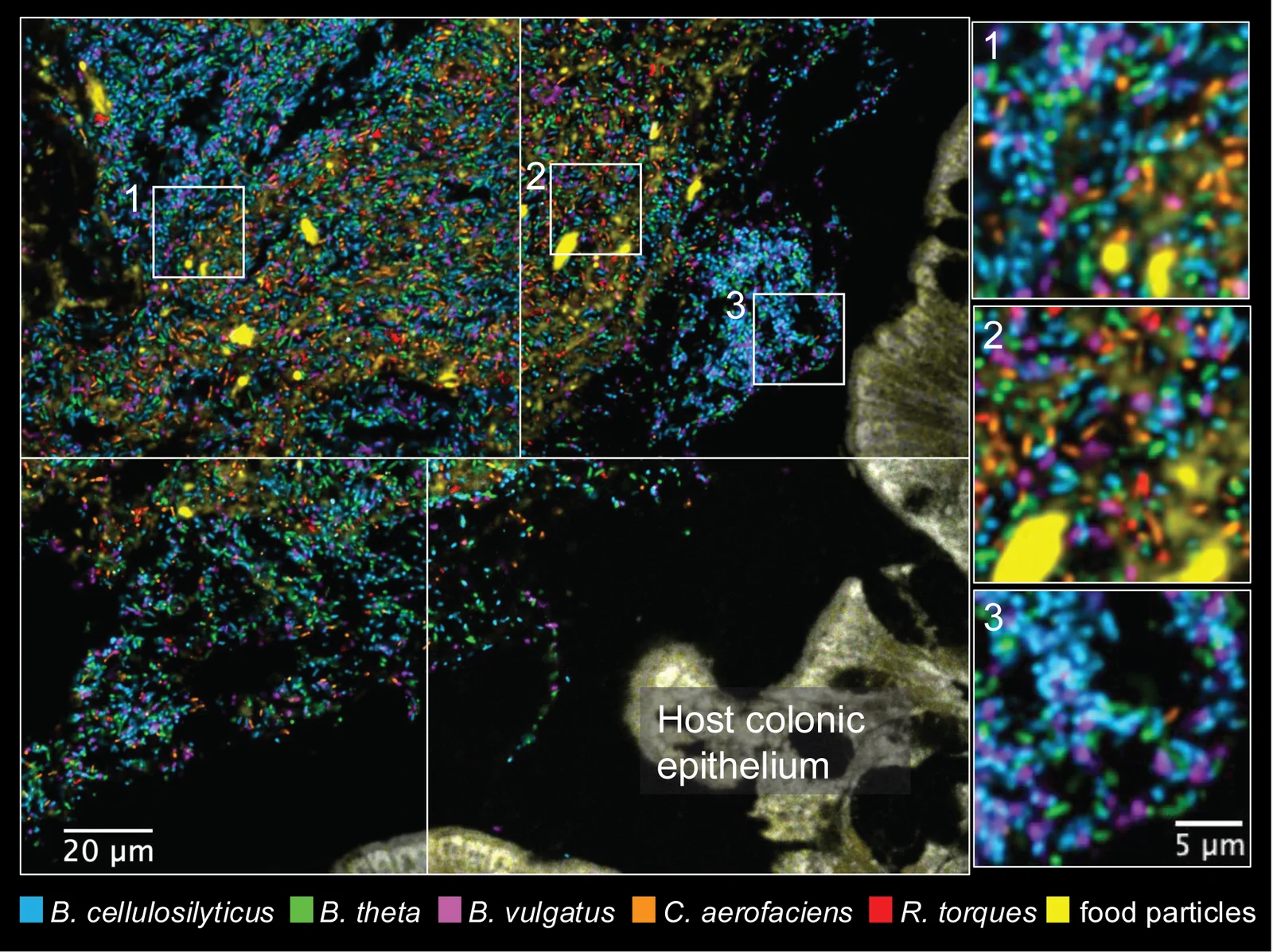
Bacteria in the gut show less spatial structure than bacteria in the mouth Bacteria in a cross-section of the mouse colon. The colonic epithelium (gray) is at right and at the bottom of the image; bacterial cells appear as colored dots, and food particles appear bright yellow. Some regions are rich in *Bacteroides* spp.; others have all five labeled cell types, but in all regions, bacteria are mixed. Image is a composite from four individual fields of view. Modified from [32].

The organization of bacteria in the gut is profoundly different from that in the mouth. In dental plaque and in biofilms on the tongue, bacteria formed filaments, chains, and single-taxon patches of various sizes; such patchiness is expected if the daughter cells produced by bacterial cell division tend to remain adjacent, forming clusters and microcolonies. In contrast, gut bacteria imaged by CLASI-FISH were spatially mixed at a single-cell level. In cross-sections of the colon of gnotobiotic mice (previously germ-free mice colonized with a set of 15 bacteria characteristic of the human gut), most bacteria were adjacent to bacteria of a different taxon, with little evident spatial structure (Figure 4 and [32]), indicating that the rate of mixing was sufficient to overcome the tendency of bacterial replication to generate single-taxon microcolonies. Bacteria reached the highest density in regions rich in mucus, both near the mucosal epithelium and within rafts of mucus in the gut lumen, and had lower density on and around particles of ingested food.

While there were no clear differences in this study in the overall distribution of bacterial species across the colon, nonetheless at micrometer scales different species of bacteria were non-randomly distributed relative to one another. *Ruminococcus torques*, a representative of the clostridia, was negatively correlated with the most abundant *Bacteroides*, *B. cellulosilyticus*, at scales of under 20 μm—which could have been a result of some antagonistic interaction between them or simply because these taxa responded differently to underlying features such as the location of mucins or ingested food.

That the distribution of bacteria in the gut responds to the distribution of mucins and ingested food was shown in a different study that used FISH to analyze bacterial spatial organization in the mouse gut [33]. This study compared mice fed a normal diet to mice fed a diet lacking in dietary fiber. The lack of fiber reduced the food available to the microbiota in the colon, and in these mice, the mucus layer in the colon was significantly thinner, allowing the microbiota to approach much closer to the epithelium. A cross-section of gut from a mouse on a standard, fiber-rich diet showed not only thicker mucus but also large swaths of bacteria of similar types, for example, *Bacteroides* in a dense aggregation near the mucus layer and Firmicutes (e.g., clostridia) in large patches in the lumen. As the probes in this study targeted broad groups of bacteria and did not discriminate between different species, this finding does not address the possibility that multiple species could be present within each broad group and mixed at the single-cell level. It does, however, show that diet and mucins affect the localization and organization of the microbiota.

How mixing occurs in the gut is the subject of a recent study considering the contents of the gut as a non-Newtonian fluid—a fluid whose viscosity changes when force is applied. Using a gnotobiotic mouse colonized with three bacterial taxa, this study found that each of the bacteria tended to be closer to other bacteria of its type, at length scales under 20 μm, than would be expected from a random distribution [34]. This proximity, they found, was the result of bacterial growth and proliferation in gut contents that behaved as a thick gel, keeping recently divided cells adjacent to one another. The nascent clusters were then broken up when the gut contents—being a non-Newtonian fluid - were liquified by pressure caused by contractions of the gut wall. Thus, in the gut as in the mouth, the spatial organization of the microbial community is the result of the proliferation and interactions of the microbiota but also the response of the microbiota to conditions established by the host.

The spatial organization of the gut microbiota has important implications for health, as the ability of bacteria to cross the mucus layer and contact the epithelium can lead to increased susceptibility to disease [35]. Further investigation of how bacteria are organized in the gut and how mucins, foods, and specific bacterial taxa affect one another may lead to breakthroughs in the management and prevention of inflammatory diseases of the digestive system.

## Social life in the sea

**Figure 5:**
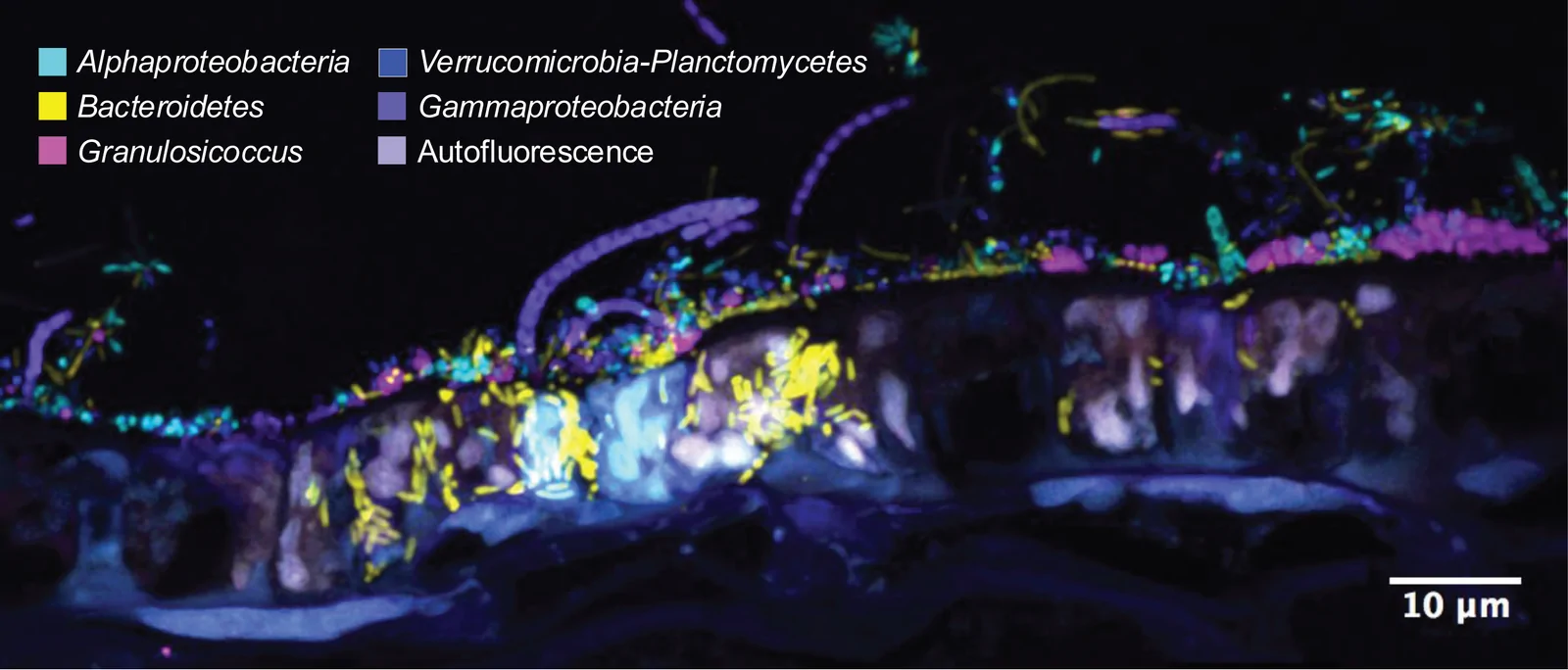
Bacteria coat the surface of a marine alga and enter its tissue Bacteria on the surface of the kelp *Nereocystis leutkeana*. A piece of the kelp blade was imaged by embedding in plastic and sectioning. A cross-sectional view shows bacteria forming a biofilm on the surface, invading the kelp tissue, and projecting into the water column. Modified from [36].

The contrast between the highly structured biofilms in the human mouth and the less organized bacterial arrangement in the gut suggests that an important factor driving spatial structure is the need to adhere to surfaces. The surfaces of marine algae resemble surfaces of the teeth and tongue in the human mouth, in that they are rich in nutrients secreted by the host but are bathed in flowing liquid. To take advantage of the nutrients, a microbe will need to adhere to the surface or burrow into the tissue to avoid being swept away by the flow. It is perhaps not surprising, therefore, that a CLASI-FISH study of bacteria on the surface of kelp revealed a clustered and spatially differentiated community similar in some ways to the community in the mouth [36]. To investigate the organization of this microbiome, kelp tissues were embedded in plastic, sectioned, and hybridized with CLASI-FISH probes. Imaging these samples (Figure 5) revealed cells from a genus of bacteria commonly associated with kelp, genus *Granulosicoccus* of phylum Pseudomonadota, forming clusters at the surface of the macroalga. Bacteria from phylum Bacteroidota (Bacteroidetes) penetrated the kelp tissue, whereas chains and filaments of other taxa projected into the water column. The surface biofilm was typically only a few micrometers thick and consisted of different bacterial taxa directly adjacent to one another, suggesting the potential for metabolic interactions of the bacteria with one another and with the host. The position of the surface biofilm, between the kelp and the water column, suggested that the bacteria might influence local water chemistry as well as modulate interactions between the kelp and other organisms, since the bacterial biofilm would be the first surface presented to any other organism encountering the kelp.

## Perspective and Outlook

CLASI-FISH has provided glimpses into the complex and intricate organization of bacterial communities, insight that naturally comes with limitations. FISH requires permeabilization of the target cells to allow nucleic acid probes to access the rRNA. Because of this need for permeabilization, FISH can be applied only to fixed samples. The development of a live-imaging version of CLASI-FISH would be an exciting innovation that would require a fundamentally different strategy for labeling. Embedding and sectioning of fixed samples improves accessibility of targets, especially in Gram-positive taxa [37], but is more time-consuming than hybridization to whole-mount samples. Additional limitations arise from the need for the investigator to have access to a microscope equipped with a spectral detector; CLASI-FISH can in principle be accomplished using sequential images taken with a standard detector, but spectral detectors, while expensive and less widely available, make image acquisition more streamlined. Development of CLASI-FISH probe sets requires not only design of numerous oligonucleotide probes but also extensive testing of the reactivity of these probes on pure cultures of known bacteria to measure the intensity of intended signals and test for the presence of unintended and unpredictable reactions of the probes with non-target taxa. These factors increase the cost and thus decrease the accessibility of CLASI-FISH experiments compared with standard FISH.

Developments in technology and data analysis can increase the number of taxa analyzed simultaneously with CLASI-FISH and related methods. One such development is a method called high phylogenetic resolution microbiome mapping by fluorescence *in situ* hybridization (HiPR-FISH), which took CLASI-FISH to its logical conclusion by imaging all 1023 possible combinations of 10 fluorophores and applied these higher-multiplexity barcodes to image samples of mouse gut and human dental plaque [38]. Combining HiPR-FISH with single-molecule FISH targeting mobile genetic elements allowed the spatial mapping of bacteriophage and plasmids within human dental plaque [39]. A method called sequential error-robust fluorescence *in situ* hybridization generated many distinct fluorescent barcodes by using multiple sequential rounds of hybridization and imaging and was used to study the distribution of microbes on the roots of *Arabidopsis* plants [40]. These methods come closer to the goal of simultaneous identification of all members of a bacterial community, but at the cost of higher experimental and computational complexity. Analyzing the results of spectral unmixing with such complex probe sets requires automated segmentation and analysis workflows that may be out of reach of many laboratories. Experimentally validating hundreds to thousands of probes by hybridization to pure cultures would be enormously time-consuming, and this validation step is therefore often omitted in these extremely high-multiplexity methods.

Application of combinatorial labeling and spectral imaging is not limited to identification of bacterial cells using rRNA-targeted probes but also can be used to identify mRNAs and other subcellular structures. Fluorescence spectral imaging has been deployed to investigate the spatial and temporal organization of organelles within eukaryotic cells [41], demonstrating the potential for detailed spatial analysis of their structure and function. Combinatorial labeling is the strategy used for multiplexed error-robust fluorescence *in situ* hybridization to carry out spatially resolved single-cell transcriptomics [42–44]. A similar strategy, named parallel sequential fluorescence *in situ* hybridization, was deployed to measure gene expression in the bacterium *Pseudomonas aeruginosa* [45]. These studies demonstrate the promise of combinatorial labeling technologies to investigate functional properties as well as spatial organization of bacterial cells. Standard FISH has been used in combination with a range of techniques to measure metabolic properties of biofilms, using pH ratiometry [46], microautoradiography [47], Raman spectroscopy [48,49], and secondary ion mass spectrometry [50]. The combination of such techniques with CLASI-FISH could provide insight into function simultaneously with structure in complex microbial communities.

These glimpses into the social lives of microbial communities are only a beginning. How communities change in disease, how they change over time, and how they are structured in different organisms or in the environment are all open questions. Any sample with an abundant, dense microbiota is an inviting target for investigation of how community organization reflects the function and interactions of microbial taxa with one another and with their environment and host.

## Summary

- Spatial organization provides clues to function and interactions in bacterial communities.
- CLASI-FISH is a strategy for identifying and localizing many different bacterial species simultaneously by microscopy.
- Dental plaque analyzed by CLASI-FISH shows a complex structure built around clusters of filaments of the bacterium *C. matruchotii*.
- Tongue dorsum biofilms analyzed by CLASI-FISH show bacteria forming patches around a core of human epithelial cells; these bacteria may play an important role in host nitrate metabolism.
- Gut bacteria show much less spatial structure.
- Bacteria on kelp, like bacteria in the mouth, bind to surfaces to avoid being swept away by fluid flow, and form spatially structured patches.

## Open Access

This article has been published open access under our Subscribe to Open programme, made possible through the support of our subscribing institutions, learn more here: https://portlandpress.com/pages/open_access_options_and_prices#conditional.

## Abbreviations

CLASI-FISH: combinatorial labeling and spectral imaging fluorescence *in situ* hybridization
FISH: fluorescence *in situ* hybridization
HiPR-FISH: high phylogenetic resolution microbiome mapping by fluorescence in situ hybridization
rRNA: ribosomal RNA
